# Supplementary material for: Impact of online negative and positive reviews on app innovation
Source: PLoS One. 2025 May 16;20(5):e0323205. doi: 10.1371/journal.pone.0323205 (PMC12083816; doi:10.1371/journal.pone.0323205)
Supplement: Attachment — Submitted filename: Response to Reviewers.doc [file pone.0323205.s002.doc]

**Response to the AE**

Dear Dr. Ayesha Maqbool，

Thank you very much for the referee report and the valuable comments on our manuscript, “*The Impact of Online reviews on App Innovation and Its contingent Effect*” (Manuscript ID: PONE-D-24-42585). We are extremely grateful to your decision on the three reviews.

We have carefully studied the comments from the review team and tried our best to respond to the issues raised by the review team. In this response letter, we first describe the major changes we have made to the manuscript in response to your comments, and we then address the individual issues raised by the three reviewers.

Comments are in italics and our responses are in normal font:

*1. The theoretical framework should integrate complementary perspectives, such as signaling theory or absorptive capacity, to enhance the analysis.*

**Response:** We have added signaling theory and revised the section on research hypotheses to strengthen the reasons behind the formulation of the hypotheses (see, the subsection “2.1 Signaling Theory” on Page 2- Page 3；the lines marked in red on Page 4；), and provided practical cases to make the hypotheses more persuasive (see, the lines 4-15 of paragraph 2 on Page 4; the lines 14-24 of paragraph 2 on Page 5; the lines 12-22 of paragraph 2 on Page 6).

*2. The dataset's scope requires a stronger justification or broader dataset should be included to address applicability concerns Figures may be redrawn or cited with proper permissions.*

**Response:** In this revision, we further provided the reasons and reference basis for the sample selection (see, the lines marked in red of subsection “3.1 Sample Selection and Data Sources” on Page 9), and re-collected the data from December 2023 to 2024 to verify the main conclusions. The regression results show that the main results remained unchanged. suggesting that our findings are robust and not subject to one specific time (see, the lines 19-25 of paragraph 2 in subsection “4.3.2 Robustness Tests” on Page 14; Model 5 in Table 3 on Page 14). In addition, we have redrawn charts and include them as part of your main manuscript.

*3. The conclusion should align more clearly with the abstract and title, reflecting key findings.*

**Response:** In this revision, we have revised the title to “Impact of online negative and positive reviews on App Innovation” and refined the abstract to highlight the key conclusions (see, the lines marked in red of subsection “Abstract” on Page 1).

*4. References must be updated to include recent and relevant studies only.*

**Response:** In this revision, we add recent and relevant studies, such as reference [3], [10] and [38] in subsection “References”.

Below we provide more detailed responses to the comments by the two reviewers

Thanks again!

**Response to Reviewer 1**

Thank you very much for your great comments and suggestions on our manuscript. We are so encouraged that you think the subject and findings are interesting and novel. We have carefully studied the comments from you and tried our best to respond to the issues raised by the review team.

Below we provide a point-to-point response to your comments:

*1. The manuscript addresses an interesting and novel topic, exploring the differential effects of online negative and positive reviews on app innovation. While the study's objective is innovative, the execution falls short, and the current version does not meet the standards for publication. Significant improvements are required in the following areas to enhance its quality and contribution.*

*The language and clarity require substantial improvement, with numerous grammatical errors and inconsistent phrasing detracting from the paper’s readability. A professional edit is essential to meet international academic standards.*

**Response:** Thank you for the constructive suggestion! Following your suggestion, this paper has been checked and corrected for proper English language, grammar, punctuation, spelling, and overall style by the highly-qualified, native English speaking editors at Native English Editing. The statement of edit is attached to Appendix I.

*2.  The theoretical framework focuses primarily on negativity bias theory, which is valid and relevant. However, incorporating complementary perspectives, such as signaling theory or absorptive capacity, could enrich the discussion and provide a broader understanding of the mechanisms at play.*
**Response:** This is a really great suggestion and thank you very much! In this revision, following your suggestion, we have added signaling theory in the section “2 Theoretical Basis and Research Hypothesis” (see, the subsection “2.1 Signaling Theory” on Page 2- Page 3) and gave a detailed explanations on the mechanisms of online negative reviews on app innovation in the subsection “2.3 Research Hypotheses” (see, the lines marked in red on Page 4).

*3. The restricted dataset and sample selection, focused only on iOS apps in China and excluding certain app categories like gaming, limits the generalizability of the findings. While this choice may reflect specific research objectives, the authors should provide a clear justification for this scope to address potential concerns about applicability.*
**Response:** Thanks for pointing this out! Following your suggestion, we further provided the reasons and reference basis for the sample selection (see, the lines marked in red of subsection “3.1 Sample Selection and Data Sources” on Page 9), and proposed that future research could separately examine the influence mechanism of online negative reviews on app innovation within the gaming industry (see, the subsection “5.4 Limitations and Future Research Directions” on Page 17).

*4. The practical implications lack actionable guidance for app developers. Providing specific strategies for leveraging online reviews in innovation would enhance the study’s relevance and impact.*
**Response:** Thanks for pointing this out! In this revision, we have rewritten the practical significance to provide actionable guidance for app developers. (see, the subsection “5.3 Practical Implications” on Page 17).

**Response to Reviewer 2**

Thank you very much for your valuable comments and very constructive suggestions in our paper. We have tried our best to revise the paper to incorporate all your suggestions and address all your concerns. Below we detail how we changed the paper in response to your comments and suggestions.

*1. Line 3-5 of “Introduction”, please add the data source.*
**Response:** Thanks for pointing this out! In this revision, we have added the data source. (see, the 3-5 lines in section “1. Introduction” on Page 1).

*2. Be careful to proof-read the whole manuscript as it looks like there are quite some grammatical errors. For example:*

*a) “Insert Figure 1 about here” might improve if replaced by “Insert Figure 1 here”;*

*b) “3.2.4 Control Variable” would be better if replaced by “3.2.4 Control Variables”;*

*c) “Due to use one period lagged data, the data period used in this study is from January 2019 to December 2019.” This sentence is poorly written and need to polish.*
**Response:** Thanks for pointing this out! In this revision, the above grammatical errors have been revised (see, the subsection “3.2.4 Control Variables” on Page 9 and the 3-4 lines in subsection “3.1 Sample Selection and Data Sources” on Page 8). Moreover, this paper has been checked and corrected for proper English language, grammar, punctuation, spelling, and overall style by the highly-qualified, native English speaking editors at Native English Editing. The statement of edit is attached to Appendix I.

*3. The sample period of your study ranges from 2018 to 2019, internet and mobile devices develop rapidly, your data is too old for readers to know the change currently. Maybe you should update your database and focus on the last 2 years.*
**Response:** Thanks for pointing this out! Following your suggestions, we re-collected the data from December 2023 to 2024 to verify the main conclusions. The regression results in Model 5 (Table 3) show that the main results remained unchanged. suggesting that our findings are robust and not subject to one specific time (see, the lines 19-25 of paragraph 2 in subsection “4.3.2 Robustness Tests” on Page 14; Model 5 in Table 3 on Page 14).

*4. I did not find the regression equation in your study, please add equations that your study related to.*
**Response:** Thanks for pointing this out! Following your suggestions, we have added a new subsection “3.2.5 Model Specification” to explain our research equation (see, the subsection “3.2.5 Model Specification” on Page 10).

*5. Each hypothesis you accept, or reject should be explained in detail. The reasons why the hypothesis is accepted or rejected this study are not adequate now.*
**Response:** Thanks for pointing this out! Following your suggestions, we have added signaling theory and revised the section on research hypotheses to strengthen the reasons behind the formulation of the hypotheses (see, the subsection “2.1 Signaling Theory” on Page 2- Page 3；the lines marked in red on Page 4；), and provided practical cases to make the hypotheses more persuasive (see, the lines 4-15 of paragraph 2 on Page 4; the lines 14-24 of paragraph 2 on Page 5; the lines 12-22 of paragraph 2 on Page 6). According to the results of the regression analysis in table2, all hypotheses have been accepted (see, the table 2 on Page 12-13).

**Response to Reviewer 3**

Thank you very much for your great comments and suggestions on our manuscript. We are so encouraged that you think the article is well-organized. We have carefully studied the comments from you and tried our best to respond to the issues raised by the review team.

Below we provide a point-to-point response to your comments:

*1.* *- While the methodological rigor is commendable, the explanations, especially for the 2SLS and instrumental variable choice, could benefit from simplification for readers less familiar with econometrics. For instance, the rationale for using the average number of reviews from similar apps as instruments could be expanded to clarify their validity further.*

**Response:** Thanks for pointing this out! Following your suggestions, in this revision, we further explain the 2SLS method (see, 1-6 lines of paragraph 2 in the subsection “4.3.1 Endogeneity” on Page 13) and strengthen the reasons for selecting the instrumental variables (see, 6-15 lines of paragraph 2 in the subsection “4.3.1 Endogeneity” on Page 13-14).

*2.* *-The terms like "mutual causality" and "omitted variables" could be briefly explained or referenced for readers unfamiliar with econometric challenges.*

**Response:** Thanks for pointing this out! Following your suggestions, we explain the two terms "mutual causality" and "omitted variables", and provided the related references (see, paragraph 1 in the subsection “4.3.1 Endogeneity” on Page 13).

*3.* *-* *Phrases such as* *"Insert Table 3 about here" disrupt the narrative. Consider integrating the description of Table 3's key results into the text, providing readers with immediate insights without needing the table.*

**Response:** Thank you for pointing it out! Following your suggestions, we delete *"Insert Table 3 about here"* and add the description of Table 3's key results into the text to make it convenient for readers to read (see, paragraph 2 in the subsection “4.3.2 Robustness Tests” on Page 14).

*4.* *-* *The section mentions that app history and platform entry positively moderate the impact of negative reviews, while app performance weakens it. This could be elaborated with examples or hypothetical scenarios to make these findings more tangible.*

**Response:** Thank you very much for your valuable comments on our manuscript! Following your suggestions, we add practical examples to make the moderating effect more persuasive and tangible (see, the lines 4-15 of paragraph 2 on Page 4; the lines 14-24 of paragraph 2 on Page 5; the lines 12-22 of paragraph 2 on Page 6).

Thanks again!

Appendix I.
