# Supplementary material for: Impact of online negative and positive reviews on app innovation
Source: PLoS One. 2025 May 16;20(5):e0323205. doi: 10.1371/journal.pone.0323205 (PMC12083816; doi:10.1371/journal.pone.0323205)
Supplement: Attachment — Submitted filename: Response_to_Reviewers_auresp_2.doc [file pone.0323205.s003.doc]

**Response to Reviewer 1**

Thank you very much for your great comments and suggestions on our manuscript. We are so encouraged that you think the manuscript has improved significantly in terms of theoretical depth, methodological rigor, and clarity. We have carefully studied the comments from you and tried our best to respond.

Below we provide a point-to-point response to your comments:

*1. Inconsistency in Data Description (Section 3.1 vs. Section 4.3.2)*

*Section 3.1 describes the dataset as 2018-2019, while Section 4.3.2 states that 2023-2024 data was collected for* *robustness checks. This inconsistency must be resolved. Revise Section 3.1 to acknowledge the use of 2023-2024 data for validation, ensuring clarity on dataset scope.*

**Response:** Thank you for the constructive suggestion! Following your suggestion, we have added the description to acknowledge the use of 2023-2024 data for robustness checks in Section 3.1 (see, the lines marked in red on Page 9).

*2.  Justification for 2023-2024 Data Inclusion*

*The manuscript does not explicitly state that 2023-2024 data was included to address concerns about data timeliness. Clarify in Section 3.1 that this dataset was collected as the latest available data to validate the findings. In Section 4.3.2, briefly* *discuss whether market conditions, platform policies, or user behavior have changed, and if not, explicitly state that the dataset remains comparable.*
**Response:** This is a really great suggestion and thank you very much! In this revision, following your suggestion, firstly, we have demonstrated that the data in 2023-2024 is collected as latest available data to address concerns about data timeliness ( see, the 1-3 lines of second paragraph in the subsection “4.3.2 Robustness Tests” on Page 14).

Secondly, we discuss the difference of market conditions, platform policies and user behavior between 2023-2024 and 2018-2019. The results show that the market environment in which apps operate has experienced more intense competition in 2023-2024. Continuous innovation has become increasingly crucial for developers, reflecting that the two sets of data from different time periods are comparable. ( see, the 3-8 lines of second paragraph in the subsection “4.3.2 Robustness Tests” on Page 14).

*3. Comparison Between 2018-2019 and 2023-2024 Data*

*The robustness test confirms that findings remain stable, but the manuscript does not compare key variable distributions between the two datasets. Provide a brief* *descriptive analysis of whether variables such as negative reviews, app performance, and innovation frequency have changed significantly over time. If no substantial differences exist, explicitly state this to reinforce the consistency and validity of the results.*
**Response:** Thanks for pointing this out! Following your suggestion, this paper presents the descriptive statistics of the latest data in table 4. Specifically, we add the descriptive analysis to compare the differences between 2018-2019 and 2023-2024 data. There are no significant or substantial differences between the two datasets ( see, the third paragraph in the subsection “4.3.2 Robustness Tests” on Page 14).

Additionally, his paper has used the two-way fixed effects model has been used to test the hypotheses, effectively controlling for the influence of time factors in the regression analysis in table 2. The results of 2018-2019 data have validity. ( see, the subsection “3.2.5 Model Specification” and table 2; the 4-6 lines of fourth paragraph in the subsection “4.3.2 Robustness Tests” on Page 15).
